# Functional Characterization of N297A, A Murine Surrogate for low-Fc Binding Anti-Human CD3 Antibodies

**DOI:** 10.1080/08820130802608238

**Published:** 2009-01-26

**Authors:** Debra T. Chao, Xiaohong Ma, Olga Li, Hyunjoo Park, Debbie Law

**Affiliations:** Research Department, PDL BioPharma, Inc. Redwood City, California, USA

**Keywords:** CD3, Monoclonal antibody, Inflammatory Bowel Disease, T cells

## Abstract

Several low- or non-FcR binding anti-human CD3 monoclonal antibodies have been under investigation for the treatment of autoimmune diseases. To model the mechanism of action of these anti-human CD3 mAbs in the murine system, an Fc-modified anti-mouse CD3 antibody (N297A) was generated. N297A exhibited similar biological effects as Fc-modified anti-human CD3 antibodies including rapid, reversible reduction in peripheral leukocyte numbers, differential modulation of activated versus resting T cells, and reduced levels of induced cytokine release compared to the non-Fc-modified parent antibody. In an in vivo model of colitis induced by adoptive transfer of IL–10-deficient cells, administration of N297A significantly reduced body weight loss. As N297A shared many functional characteristics of non-FcR binding anti-human CD3 mAbs both in vitro and in vivo, it provides a means to model the mechanisms of action of Fc-modified anti-human CD3 antibodies in mouse.

## INTRODUCTION

In 1986, the murine anti-human CD3ɛ monoclonal antibody (mAb), OKT3 (muromonab-CD3), was approved for the treatment of steroid-resistant renal transplantation rejection (Thistlethwaite et al., 1988). OKT3 proved to be a strong immunosuppressive agent due to its broad reactivity with all T cells. However, the induction of both human anti-mouse antibodies to OKT3 and severe cytokine release syndrome (CRS) resulting from the potent agonistic activity of this mAb have limited its use (Gleixner et al., 1991; Sgro, 1995; Thistlethwaite et al., 1988).

Attempts to produce anti-human CD3 mAbs without the side effects of OKT3 have generated mAbs with improved tolerability and safety characteristics (Carpenter et al., 2002; Cole et al., 1999; Norman et al., 2000). A common feature of these improved mAbs is the reduction of the murine amino acids with those commonly found in human immunoglobulins (humanization), reducing the level of immunogenicity. The second common feature of these antibodies is the modification of the Fc portion of the antibodies to reduce FcR binding and therefore reduce side effects associated with cross-linking of CD3 via an Fc-dependent mechanism.

Representatives of this novel class of next generation anti-CD3 antibodies include visilizumab (HuM291; NUVION®), teplizumab (hOKT3γ1-Ala-Ala), and ChAglyCD3 (TRX4). Each of these mAbs has been under evaluation in clinical trials for T cell mediated autoimmune indications including graft versushost disease, ulcerative colitis, Crohn's disease and type I diabetes (Carpenter et al., 2002; Herold et al., 2003; Keymeulen et al., 2005; Plevy et al., 2007; Targan S, 2005; Vexler and Woo, 2006; Woodle et al., 1998). The Fc-modified anti-human CD3 mAbs have shown promise in a number of these trials and have an improved safety profile, compared to the original OKT3 therapy. Common adverse events in the recent clinical trials include mild to moderate flu-like symptoms, rash, and transient symptoms of Epstein-Barr viral mononucleosis (Carpenter et al., 2002; Keymeulen et al., 2005). The flu-like symptoms with anti-CD3 therapy are a class phenomenon and attributable to cytokine release which, although dramatically reduced by the Fc modifications, still occurs when the anti-CD3 mAbs bind to T cells (Alegre et al., 1991; Ferran et al., 1990; Vossen et al., 1995). Another common characteristic of these Fc-modified mAbs is a rapid reduction of peripheral blood lymphocytes following the initial dosing, which is a useful pharmacodynamic marker for the mAb activity (Bisikirska et al., 2005; Carpenter et al., 2002; Hommes et al., 2005; Hsu et al., 1999). However, the relationship of the change in peripheral blood T cell count and clinical response remains to be elucidated.

Potential mechanisms of action of antibody therapies are often evaluated using animal models of human disease. However, the restricted binding of the anti-human CD3 mAb to human and chimpanzee has prevented studies in small animal models as well as in non-human primates. Use of Fc-modified anti-murine CD3 antibodies that can act as surrogates for their human counterparts has therefore been crucial for these mechanistic studies. Important early data came from studies using a hamster anti-mouse CD3 antibody (145.2C11). Data generated using this mAb allowed a better understanding of the role/function of the Fc region of anti-CD3 antibodies. Subsequent studies with F(ab′)2 fragments of 145._2_C11 demonstrated that the Fc receptor binding capacity of the mAb could be dissociated from efficacy in animal models (Alegre et al., 1995; Yu and Anasetti, 2000; Yu et al., 2001).

Since the potent mitogenic activity of OKT3 was due in large part to Fc-dependent interactions that allowed for cross-linking of the CD3 complex, the fact that efficacy could be retained in animal models with a non-Fc containing mAb helped pave the way for the anti-human Fc-modified therapies. While the 145.2C11 mAb, and fragments there of, have been invaluable in furthering our understanding of an anti-CD3 therapeutic approach, issues with the production of large quantities of pure F(ab′)_2_ fragments prompted us to search for an alternative anti-mouse CD3 analogue.

In this study, we detail the generation of an intact, Fc-modified antimouse CD3 mAb (N297A), which shares many of the functional characteristics of the Fc-modified, low mitogenic anti-human CD3 antibodies. In addition, N297A can alleviate clinical signs of disease in a murine model of colitis. Together, these in vitro and in vivo data suggest that we have generated an Fc-modified anti-CD3 mAb surrogate that should prove useful in the further dissection of potential mechanisms of action of Fc-modified anti-CD3 mAb in clinical development.

## MATERIAL AND METHODS

### Generation of an Fc-Modified Anti-Murine CD3 mAb (N297A)

The CDR sequence from hamster anti-mouse CD3 mAb (145.2C11) was combined with the murine IgG1 backbone to make a chimeric hamster/mouse mAb, 145.2C11.mIgG1.wt (WT). A variant containing a modified Fc region was made by an alanine substitution of murine IgG1 heavy chain at amino acid N297 in the CH2 region using site directed mutagenesis PCR with the following two primers: 5′ GCAACC CCGGGA GGAGCA GTTCGCCAGCA 3′ and 5′ TATTGC GGGGTC CCAAGG CAGTGC 3′. SP2/0 cells were then co-transfected with the heavy chain expressing vectors and p145.2C11-mouse Cl under mycophenolic acid selection.

Positive clones were chosen based on high mouse IgG1 production detected by ELISA and binding to EΔC cells (mouse T cells expressing CD3ɛ) by flow cytometry. An equivalent negative control mAb (NCNS-1) was generated by replacing the CDR3 variable region of N297A with non-CD3 binding hamster antibody sequences; this control mAb was confirmed to have lost the ability to bind to murine CD3ɛon EΔC cells.

### Fc Receptor Binding Assay

Expression of Fcγreceptors CD16/32 and CD64 on RAW 264.7 mouse macrophage cell line were confirmed by flow cytometry. Both RAW264.7 and 3T12 cell lines were purchased from ATCC (Bethesda, MD). Control mouse IgG2a, IgG2b (both against VP7 of blue tongue virus; #CRL-1875 and 1877), and IgG1 (anti-TNP mAb; #TIB-191) antibodies were purified from hybridoma cell lines purchased from ATCC. 5 × 10^5^ cells were mixed with serial dilution of primary antibody and incubated at 4°C for 20 minutes. Cells were washed once with phosphate-buffered saline (PBS) and then incubated with 2.5 μg/ml of APC conjugated goat anti-mouse IgG, F(ab′)_2_ fragment specific antibody (Jackson ImmnoResearch, West Grove, PA) at 4°C for 20 minutes. Samples were washed in PBS and resuspended in 300 μl of FACS buffer and analyzed by flow cytometer (FACSCalibur, BD, San Jose, CA).

### In vitro Cell-Based Assays

For plate bound antibody stimulation, antibodies were diluted in PBS and loaded in triplicate into 96-well plates. Plates were left overnight at 4°C or for 1.5 hours at 37°C to ensure coating of antibodies. Plates were washed twice with PBS, and 2 × 10^5^ cells were loaded per well in 100 μl of T cell media (RPMI /10% FCS /15 mM HEPES/2 mM L-glutamine /1 mM sodium pyruvate /50 μM 2-mercaptoethanol with penicillin/streptomycin). For soluble antibody stimulation, 2 × 10^5^ cells were seeded per well in 100 μl of T cell media. Anti-mouse CD3 antibodies were diluted in PBS and then added to the cells and cultured for 48 hours. Then, 10 μl of the cell proliferation indicator AlamarBlue® (Biosource, Camarillo, CA) were added per well, and plates were incubated at 37°C in a 5% CO2 incubator for 4 hours. Plates were read using a 96-well fluorescence plate reader (SpectraMax GeminiXS, Molecular Devices, Sunnyvale, CA) with excitation at 530 nm and emission at 590 nm.

For apoptosis assays, spleen cells (2 × 10^6^ /ml in T cell media) were activated with 1 μg/ml of concanavalin A (Con A) (Sigma, St. Louis, MO) for 96 hours.

Viable leukocytes were recovered using Ficoll-Paque (Amersham Bioscience, Piscataway, NJ) and resuspended at 2 × 10^6^ cells/ml in T cell media supplemented with mouse IL-2 (200 U/ml). Antibodies and cells were incubated for 24 hours at 37°C. Apoptotic cells were then stained using TACS Annexin V/PI kit (R&D, Minneapolis, MN). Caspase activity was measured by adding Caspase-Glo® 3/7 Substrate (Promega, Madison, WI) and incubating at RT for 30 minutes before measuring luminescence (Perkin Elmer, Waltham, MA).

### Animals

IL-10.NOD-deficient mice (stock #004266) were obtained from The Jackson Laboratory (Bar Harbor, ME) and bred in-house to generate sufficient mice for the experiments. Six-week-old BALB/c and C57/B6 mice were purchased from Taconics (Germantown, NY); NOD/C.B-17/scid mice (stock #001303) were from The Jackson Laboratory. Homozygous TCR transgenic male mice DO11.10 were purchased from Jackson Laboratory (stock #003303), and bred with BALB/c females to produce heterozygous transgenic mice that were used for the experiments. Animal studies were performed following the Guidelines for Ethical Conduct in the Care of Use of Animals with the approval of the PDL BioPharma Institutional Animal Care Use Committee.

### Monitoring of Peripheral Blood and Detection of Cytokines

Samples were run on a hematology analyzer (Hemavet 950, Drew Scientific, Waterbury, CT) to obtain a complete blood count. Quantification of blood cell subsets from 50 μl of whole blood was performed using TruCOUNT assays with the following mAbs: APC anti-CD4, FITC anti-CD8, PE anti-CD25 and PerCP anti-CD45 (BD BioScience, San Jose, CA). To measure cytokine and chemokine concentrations in plasma, 30 μl of each plasma sample was processed using a 22-plex LINCOplex kit (Linco Research, St.Charles, MO) following the manufacturer's instructions.

### TCR Transgenic Mice Adoptive Transfer

Spleens were harvested from heterozygous DO11.10 TCR transgenic female mice (8–12 weeks old). Single cell suspensions were prepared and stimulated with 0.3 μM of OVA peptide for 72 hours in culture. Activation of CD4^+^ T cells was verified by CD25 expression. Viable cells were isolated using Ficoll and transferred intravenously (i.v.) into BALB/c female mice (2 × 10^7^ cells per mouse). Then, 24 hours after cell transfer, the mice were dosed i.v. with mAb. Mice were sacrificed 18 hours after mAb treatment, and organs were harvested to generate single cell suspensions for flow analysis. The mAb KJ1–26 was used to identify the proportion of DO11.10 transgenic CD4^+^ T cells.

### IL-10 KO Adoptive Transfer IBD Model

Spleens were harvested from IL-10.NOD-deficient male mice that were older than 10 weeks. CD4^+^ T cells were isolated using the CD4 Dynabeads kit (Invitrogen, Carlsbad, CA). The magnetic beads were removed from the isolated cells with DETACHaBEAD®Mouse CD4 prior to injection. CD4^+^ T cell purity was checked by FACS and usually exceeded 95%. Two million CD4^+^ T cells were injected i.v. into the tail vein of each NOD/CB.17/scid male mouse. The mice were checked for clinical symptoms, such as ruffled coat and hutched posture, and weighed 3 times per week. Colons were harvested and fixed in 10% buffered formalin for histology at study termination. Slides were stained with hematoxylin and eosin for morphological analysis and then reviewed by a certified veterinary pathologist.

## RESULTS

### Biological Characteristics of N297A in vitro

Several anti-human CD3 antibodies have been engineered to reduce their binding to Fc gamma (Fcγ) receptors. In order to generate a low-FcγR binding anti-mouse CD3 antibody similar to the human counterparts, a hamster/mouse chimeric antibody with an alanine substitution at amino acid 297 in the CH2 region of mouse IgG1 was made as described above. Since IgG binding to CD16 (FcγRI) had the highest affinity compared with other Fcγ receptors, CHO cells were transfected with mouse CD16 and used to analyze FcγR binding (Ravetch and Bolland, 2001).

**Figure 1:**
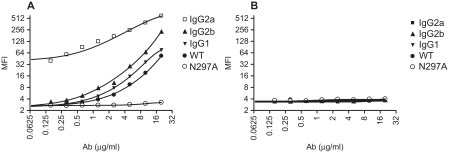
N297A did not bind to Fc gamma receptors. (A) RAW264.7, a mouse macrophage cell line which highly expresses Fcγ receptors, and (B) 3T12, a non-Fcγ receptor-expressing mouse cell line, were used to analyze binding of antibodies. Serial dilution of mouse antibodies IgG2a (□), IgG2b (▴), IgG1 (▾), WT (•) and N297A (○) were incubated with the cells for 20 minutes on ice. After washing, samples were incubated with secondary APC-conjugated goat anti-mouse IgG, F(ab′)_2_ fragment specific antibody (2.5 μg/ml) for detection by flow cytometry.

As expected, binding of N297A to mouse CD16 expressed on CHO cells was substantially reduced compared with non-Fc-modified wild-type IgG1 antibody (WT) (secondary mAb only MFI: 8.8; N297A MFI: 13.3; WT MFI: 127.3). In addition, binding to other activating Fcγ receptors was also tested using RAW 264.7, a mouse macrophage cell line which endogenously expresses CD16, CD32, and CD64 (FcγRI, IIB, III). Control mouse antibodies that did not bind to mammalian antigens were used to detect FcγR binding on RAW cells. Different isotypes of mouse IgG antibodies typically display various affinities to Fcγ receptors (Nimmerjahn and Ravetch, 2007). As expected, mouse IgG2a showed the strongest binding, followed by mouse IgG2b, and lastly mouse IgG1 (Figure 1A).

Importantly, non-Fc-modified anti-CD3 IgG1 (WT) antibody had similar binding pattern as the control mouse IgG1 antibody, whereas the mutant N297A had no detectable FcγR binding. None of the antibodies demonstrated detectable binding to 3T12, a non-FcγR expressing mouse fibroblast cell line (Fig. 1B). In addition, neither WT nor N297A mediated complement fixation in vitro with purified naïve spleen T cells as targets while an IgG3 variant of the same antibody was able to fix complement (data not shown). These data confirmed that the mutant N297A had reduced FcγR binding compared to its original antibody (WT).

**Figure 2:**
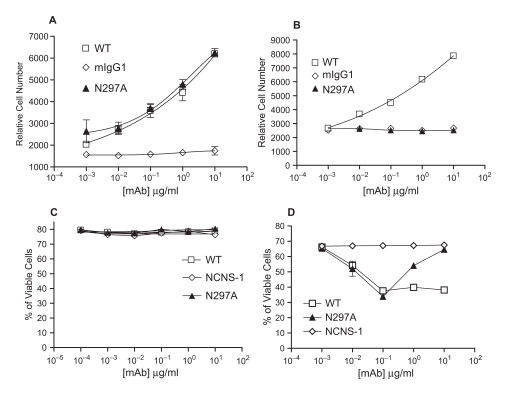
Soluble N297A did not induce proliferation but induced apoptosis in activated T cells. Spleen cells from BALB/c mice were incubated with either plate-bound (A) or soluble (B) mAbs for 48 hours. Proliferation was determined following incubation with alamarBlue. WT (□), N297A (▴), or mIgG1 isotype control (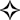). (C+D) Spleen cells from BALB/c mice were treated with WT (□), N297A (▴), or NCNS-1 negative control (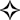) mAb for 24 hours with either no prior activation (C) or prior stimulation with Con A for 96 hours (D). Apoptotic cells were then stained using TACS Annexin V/PI kit. Cells that did not stain for Annexin V or PI were calculated and shown as % of viable cells. Triplicate samples for each condition were analyzed and average values are shown in the graphs.

To examine the mitogenic potential of N297A on spleen cells, N297A was immobilized in the wells or used in soluble form. Plate-bound N297A induced a similar level of proliferation of murine spleen cells as chimeric 145.2C11.mIgG1.wt (WT) (Figure 2A). However, while WT stimulated significant proliferation of spleen cells when added in solution, the soluble N297A mAb was unable to stimulate detectable proliferation in BALB/c spleen cells (Figure 2B). The inability of soluble N297A to induce proliferation was also observed when spleen cells from multiple mouse strains were tested (C57/B6, DBA/J1 and NOD/Ltj) (data not shown).

It has been reported that anti-human CD3 mAbs induce apoptosis of activated human T cells in vitro (Carpenter et al., 2000). Thus, we examined the apoptotic effect of N297A on activated versus resting mouse spleen cells. Neither the WT nor N297A induced cell death when incubated with resting spleen cells (Figure 2C). However, both N297A and WT were capable of inducing significant cell death in activated spleen cells to a similar maximum level (Figure 2D). Whereas WT exhibited a typical dose-response curve, the N297A variant demonstrated a maximal effect at 100 ng/ml, with higher doses becoming less effective. This prozone effect has also been observed with Fc-modified anti-human CD3 antibodies (Carpenter et al., 2000).

To confirm that the cell death induced by N297A was via apoptosis, we evaluated the activities of caspase 3 and caspase 7 (standard markers of apoptotic death). Increased caspase activity was indeed observed, with maximal activity occurring at concentrations of mAb equivalent to those that gave maximal killing in the annexin V/PI assay. Again, higher concentrations of N297A showed a prozone effect (data not shown). Taken together these data indicate that N297A is a relevant murine surrogate for Fc-modified anti-human CD3 mAbs with respect to its characteristics in vitro.

### N297A Treatment Results in the Transient Reduction of Peripheral Blood Lymphocytes and Reduced Cytokine Release in vivo

**Figure 3:**
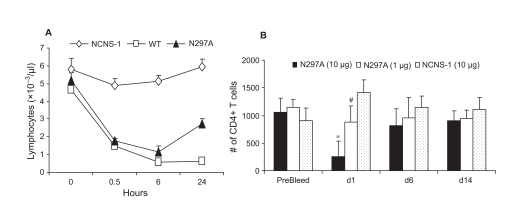
N297A caused a rapid and transient disappearance of blood lymphocytes. (A) C57/B6 mice were injected i.v. with 10 μg of N297A (▴), WT (□) or NCNS1 (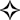) mAb. Blood samples from 5 mice per group were collected in EDTA coated tubes at indicated time points for total lymphocyte counts. (B) Different dose levels of N297A (10 μg, black bars, or 1 μg, white bars) or NCNS-1 (dotted bars) were injected i.v. into naïve C57/B6 mice at day 0 (n=5). The mice were bled at the indicated time point, and CD4^+^ T cells were quantified by flow cytometry using TruCOUNT reagents; counts per μl are shown. An unpaired two-tailed *t*-test was used for statistical analysis. *p=0.0002 for N297A (10 μg) vs. NCNS-1; # p=0.019 for N297A (1 μg) vs. NCNS-1 at day 1. The differences at other time points were not statistically significant.

Treatment of patients with Fc-modified anti-CD3 mAbs leads to a transient decrease in blood leukocyte counts (Norman et al., 2000). To determine if this decrease in peripheral blood cells was also mirrored in mice treated with N297A, mice were dosed either with N297A or control antibodies and bled at various times thereafter. Hemavet and TruCOUNT analyses were used to follow general blood cell populations as well as specific subpopulations of T cells (CD4^+^ and CD8^+^ subsets). A reduction in the number of blood lymphocytes was detected in samples from both WT and N297A (10 μg/mouse) treated BALB/c mice as early as 30 minutes post-i.v. injection (Figure 3A). The blood lymphocytes counts remained low for 24 hours. Both WT- and N297A-induced blood lymphocyte reduction were also consistently observed in other strains of mice, including C57/B6, DAB/J, CD-1 and SJL (data not shown). The reduction of T cells associated with N297A treatment was transient as has been observed in humans treated with Fc-modified anti-CD3 mAb. Both CD4^+^ and CD8^+^ peripheral T cell numbers returned to baseline within 6 days (Figure 3B).

Even with modifications of the FcγR binding regions of humanized antihuman CD3 mAb, elevated serum cytokine levels have still been observed after the initial dose (Herold et al., 2003; Keymeulen et al., 2005; Norman et al., 2000). To assess whether N297A induced cytokine release in vivo, naïve BALB/c mice were injected intraperitoneally (i.p.) with antibodies, and plasma samples were obtained at 2, 6, and 24 hours post injection. The levels of cytokines and chemokines were then measured using multiplex analysis. As expected, WT strongly induced the release of many cytokines and chemokines in the blood by the 2 hour time point. By 6 hours, levels of most cytokines had declined but were still higher than baseline. By comparison, N297A-treated mice had greatly reduced but still detectable levels of cytokines at 2 hours; these levels were maintained or increased by 6 hours (Table 1). For example, N297A induced IL-2, IL-6 and KC in the blood but at 10–40 fold lower levels than observed with WT mAb. In contrast, at 6 hours, IP-10 levels were similar between N297A- and WT-treated animals.

**Table 1:** N297A treatment induces reduced but detectable levels of cytokines and chemokines compared with WT.

	2 hours			6 hours		
		
	mIgG1	WT	N297A	mIgG1	WT	N297A
IL-1α	38	399	67	49	1358	715
IL-2	4	4678	118	4	740	168
IL-4	0	1182	11	0	15	3
IL-5	13	102	33	18	1135	508
IL-6	38	7776	425	78	998	532
IL-10	24	348	46	16	695	74
IL-12	19	55	21	14	1172	67
IL-13	45	461	51	41	181	141
IFNγ	2	740	6	1	1788	8
TNFα	10	328	37	9	115	67
G-CSF	275	4533	824	296	16506	9999
GM-CSF	8	898	32	0	76	83
IP-10	442	2571	1196	145	1069	1302
KC	22	4173	399	15	656	556
MCP-1	19	6546	175	14	2626	1129
MIP-1α	367	1437	116	74	277	324

Units are pg/ml.

Five BALB/c mice per group were injected i.p. with indicated antibody and bled after 2 or 6 hours for analysis.

The reduced level of cytokines induced by N297A treatment was consistently seen in multiple strains of mice including BALB/c, C57/B6 and IL-10.NOD deficient mice (data not shown). The induction of cytokines and chemokines by both versions of the anti-murine CD3mAbs was transient. By 24 hours post-injection, the cytokine/chemokine levels returned to base line (i.e., levels observed in control mAb treated mice; data not shown). Interestingly, there was no additional cytokine release observed when the mice were treated with a second dose of N297A. This phenomenom was also observed in human patients upon treatment with a second dose of Fc-modified antihuman CD3 mAb. N297A, like the Fc-modified anti-human CD3 mAbs, therefore induced a reduced level of cytokine release in vivo compared to WT after initial antibody treatment.

### N297A Preferentially Modulates Activated T Cells in vivo

In order to better understand the biological relevance of N297A-induced apoptosis in activated but not resting T cells in vitro, we activated OVA Ag specific T cells from DO11.10 transgenic mice for an adoptive transfer study. CD4^+^ T cells from DO11.10 mice were activated in culture with OVA peptide and transferred into naïve BALB/c mice. One day after the cells were transferred, the mice were dosed i.v. with 10 μg of mAb. Mice were sacrificed the next day, and organs were harvested for analysis. Activated DO11.10 T cells were identified using the KJ1–26 antibody which recognizes the specific TCR expressed by these cells.

**Figure 4:**
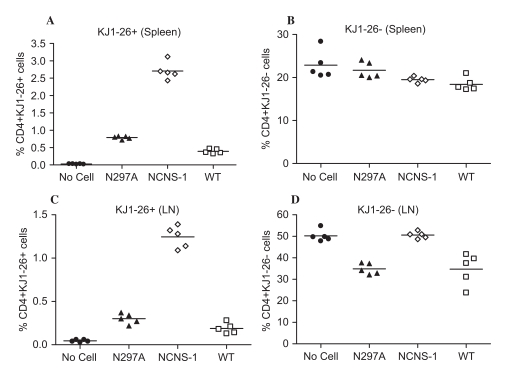
Anti-CD3 mAbs preferentially modulate activated T cells in vivo. Spleen cells from DO11.10 TCR transgenic mice were activated in vitro for 72 hours with OVA peptide prior to transfer to BALB/c mice. 24 hours after cell transfer, mice were dosed with 10 μg antibody i.v. (n=5 per group), and then sacrificed 18 hours after antibody treatment. Spleen and peripheral lymph nodes were harvested for analysis. Levels of pre-activated CD4+ donor cells (KJ1–26^+^) from spleen (A) or lymph node (C) were greatly reduced by treatment with both anti-CD3 mAbs. Levels of host CD4+ cells (KJ1–26-) showed little change in the spleen (B) or a moderate decrease in lymph node (D) following treatment of N297A (▴), NCNS-1 (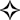) and WT (□) mAb. Mice that did not receive any OVA-activated cells (No cells •) were used as control.

When animals were treated with N297A or WT mAb, both host and donor populations of T cells disappeared from the blood (as has been consistently observed with anti-CD3 mAbs). However, there was a clear difference between the resting host T cells and activated donor T cells in the secondary lymphoid organs. Thus, 80 to 90% of OVA-activated T cells were eliminated in the spleens of N297A- and WT-treated mice compared to the negative control group (Figure 4A, p < 0.0001 for NCNS-1 vs. N297A or WT). Meanwhile, there was less effect of these antibodies on the host T cells in the spleen (Figure 4B; p > 0.01 for NCNS-1 vs. N297A or WT). Similar results were observed in the lymph nodes where N297A and WT treatment led to a reduction of over 80% of activated OVA-specific T cells (Figure 4C) but only about 30% of host T cells (Figure 4D). These data demonstrate that N297A differentially affected activated T cells compared to resting T cells in vivo.

### Efficacy of N297A in an Adoptive Transfer Model of IBD

**Figure 5:**
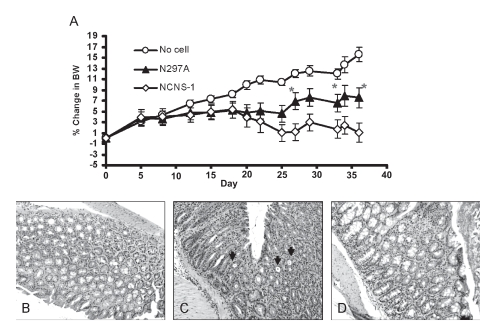
N297A treatment slowed bodyweight loss in the IL-10-deficient adoptive transfer IBD model. CD4^+^ T cells from IL-10 deficient mice were purified and transferred into NOD/scid mice to induce IBD. The antibody treatments were started at disease onset when the mice started losing body weight. (A) Mice were treated i.v. with 10 μg of N297A (n=9, ▴) or NCNS-1 (n=9, 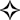) 2 to 3 times a week for a total of six doses starting at day 20. No cell control group (❍) did not receive any T cells and thus continued to grow and gain body weight (n=6). Percent change in body weight (BW) was calculated by subtracting body weight on day of measurement from ody weight on day 0 and divided by the initial body weight. Statistical significance was assessed at each time point using an unpaired, two-tailed t-test to derive the p values between N297A and NCNS-1 groups (*p < 0.05). (B-D) Representative histological images of the IBD study. (B) Normal colon of control mice that did not receive T cells. Colons of IBD mice that were treated with 10 μg of NCNS-1 (C) or N297A (D). The colons were harvested at the end of study and stained with H/E for morphology. The slides were reviewed by a certified pathologist. Severe inflammation and crypts filled with neutrophils were observed (arrows) in the colons of most NCNS-1 treated mice.

Next, we examined the therapeutic potential of N297A in a murine model of colitis. The IL-10 KO transfer model is characterized by a loss of body weight starting approximately three weeks after transfer of the IL-10-deficient CD4^+^ T cells into NOD/scid recipients. As the disease progresses, these mice exhibit signs of ill-health including ruffled coats, lethargic appearance, loose stools and significant body weight loss. Histological evaluation of colons showed that while the mice that did not receive any IL-10 deficient T cells had normal colons (Figure 5B), animals that did receive cells had significant inflammation of the colon with lymphocyte infiltration, epithelial hyperplasia, loss of epithelial integrity, and loss of goblet cells; this was apparent at 2 to 3 weeks post cell transfer (Figure 5C).

Antibody treatment was initiated in this IL-10 KO transfer model at the onset of disease symptoms with animals receiving total of 6 doses (10 μg per dose). Reduction of blood CD4^+^ T cells in the diseased mice was observed after the first dose of N297A (data not shown). While control mice that did not undergo the cell transfer procedure continued to gain weight, the mice that received IL-10 deficient CD4^+^ T cells started to show signs of disease and body weight loss. Treatment with N297A resulted in significantly less weight loss, and animals did not show the ruffled fur and hunched posture that was observed in mice treated with NCNS-1 (Figure 5A).

Although the difference in body weight was not large, animals in the N297A treated group had more stabilized body weights compared with control treated animals and the data was consistent in three separate studies (group size = 9 to 10 in each study). Therefore, N297A treatment appeared to slow down disease progression and was able to reverse the body weight loss in this murine IBD model.

Histological sections of these colons were also analyzed by a certified veterinary pathologist in a blinded fashion for the study. Although there was large animal-to-animal variation of the histological analysis of the colon, many colons from NCNS-1 treated mice had severe inflammation, hyperplasia and presence of crypts with infiltration of neutrophils, whereas colons from some of the N297A treated mice had less lymphoid infiltration and more intact goblet cells in their colons (Figure 5D).

## DISCUSSION

In the past decade, several anti-human CD3 mAbs have been engineered with the aim of reducing the side-effects observed with the original murine anti-CD3 therapeutic, OKT3 (Carpenter et al., 2002; Jacobsohn, 2002; Keymeulen et al., 2005; Trajkovic, 2002). These next generation anti-CD3 mAbs have been modified in two main ways. First, they were humanized to reduce human anti-mouse antibody (HAMA) responses. Second, they have been modified in the Fc region to diminish the antibody interaction with Fc receptors.

With OKT3, crosslinking of the antibody via interactions with FcRs on leukocytes was responsible, at least in part, for the potent stimulatory effects of the antibody that resulted in a high level of cytokine release. The Fc modifications in the next generation anti-CD3 mAbs result in a reduced mitogenic potential and abrogated cytokine release.

These modified anti-CD3 mAbs are being evaluated in a number of autoimmune disease settings. However, their lack of cross-reactivity to CD3 in lower species has limited mechanistic studies of these antibodies in preclinical animal models.

To address this issue, we engineered an anti-mouse CD3 mAb to exhibit many of the in vitro and in vivo characteristics of the anti-human CD3 mAb visilizumab. We first chimerized the hamster anti-mouse CD3 mAb 145.2C11 to a mouse IgG1 backbone and then introduced a mutation that effectively abrogated interactions with FcγRs. In the present study we demonstrated that this antibody, termed N297A, had many functional characteristics in common with visilizumab, including the ability to induce apoptosis in activated but not resting T cells, the ability to induce the rapid, transient disappearance of leukocytes from the periphery, and reduced binding to FcγRs compared to non-Fcmodified parental antibodies with a resulting reduction in the levels of cytokines released into the blood stream.

In clinical trials with the Fc-modified anti-human CD3 mAbs, mild to moderate levels of cytokines were detected between 1 to 8 hours post first dose (Carpenter et al., 2005; Herold et al., 2005; Keymeulen et al., 2005; Targan S, 2005; Vexler and Woo, 2006). Similarly, we demonstrated that cytokines were significantly reduced but still detectable upon treatment with N297A compared to WT mAb in our study. Previous reports using other “non-mitogenic” anti-mouse CD3 mAbs had suggested that these mAbs may not induce any systemic cytokine release (Li et al., 2006; Ochi et al., 2006). However, the time point that these groups used for the assessment of cytokine release was at 24 hours post-dose. In our study, circulating cytokine levels were back to baseline at this time point.

In addition, N297A was shown to have therapeutic activity in a murine colitis model induced by adoptive transfer of IL-10 deficient CD4^+^ T cells. IL-10 deficient mice develop spontaneous colitis due to immune imbalance and lack of negative regulation (Davidson et al., 1996). When cells from these mice are transferred to NOD/scid recipients, the pathogenic activated CD4^+^ T cells infiltrate the colons and cause severe colitis accompanied by significant body weight loss. N297A treatment at disease onset reversed the bodyweight loss associated with colitis. In contrast to the well-tolerated N297A treatment, the WT mAb was poorly tolerated and indeed was toxic to these diseased animals, with most mice dying within 24 hours of the initial dose (data not shown).

While N297A bears many characteristics of Fc-modified anti-human CD3 mAbs, one difference in vitro is in the ability of some mAbs to stimulate low levels of leukocyte proliferation. For example, visilizumab can elicit low levels of mitogenicity in human PBMCs although a large donor-to-donor variation is observed (Cole et al., 1997). In contrast, soluble N297A was unable to stimulate any detectable proliferation in spleen cells from multiple strains of mice. The difference could be explained partially by the differential antigen exposure since most naive mice have been exposed to few if any antigens, whereas all human donors have been exposed to numerous environmental antigens.

Taken together, these data suggest that N297A will be a useful tool to further our understanding of the mechanism of action of Fc-modified antihuman CD3 mAbs. Indeed recent work from Bisikriska et al. has suggested that the induction of regulatory T cells may be one important mechanism by which Fc-modified anti-human CD3 mAbs exert their therapeutic benefit (Bisikirska et al., 2005). We were unable to evaluate whether N297A could induce regulatory T cells in our adoptive transfer model, as the host mice are immunodeficient and IL-10 may play a role in the T regulatory function (Endharti et al., 2005; Gangi et al., 2005; Zhang et al., 2004). However, our in vivo evaluation of N297A in a transgenic mouse system demonstrated that the Fc-modified anti-CD3 mAbs preferentially impacted activated T cells while having limited, if any, effect on resting T cell numbers. These data are consistent with those seen in vitro and suggest that Fcmodified anti-CD3 mAbs maybe ideally suited for targeting pathogenic “activated” T cells in vivo.
